# Mesenchymal stem cells enhance tumorigenic properties of human glioblastoma through independent cell-cell communication mechanisms

**DOI:** 10.18632/oncotarget.25346

**Published:** 2018-05-15

**Authors:** Carolina Oliveira Rodini, Patrícia Benites Gonçalves da Silva, Amanda Faria Assoni, Valdemir Melechco Carvalho, Oswaldo Keith Okamoto

**Affiliations:** ^1^ Centro de Pesquisa sobre o Genoma Humano e Células-Tronco, Departamento de Genética e Biologia Evolutiva, Instituto de Biociências, Universidade de São Paulo, Cidade Universitária, São Paulo, SP, Brazil; ^2^ Fleury Group, São Paulo, Jabaquara, São Paulo, SP, Brazil; ^3^ Departamento de Hemoterapia e Terapia Celular, Hospital Israelita Albert Einstein, São Paulo, SP, Brazil

**Keywords:** glioblastoma, tumor microenvironment, mesenchymal stem cells, aggressiveness, secretome

## Abstract

Mesenchymal stem cells (MSC) display tumor tropism and have been addressed as vehicles for delivery of anti-cancer agents. As cellular components of the tumor microenvironment, MSC also influence tumor progression. However, the contribution of MSC in brain cancer is not well understood since either oncogenic or tumor suppressor effects have been reported for these cells. Here, MSC were found capable of stimulating human Glioblastoma (GBM) cell proliferation through a paracrine effect mediated by TGFB1. Moreover, when in direct cell-cell contact with GBM cells, MSC elicited an increased proliferative and invasive tumor cell behavior under 3D conditions, as well as accelerated tumor development in nude mice, independently of paracrine TGFB1. A secretome profiling of MSC-GBM co-cultures identified 126 differentially expressed proteins and 10 proteins exclusively detected under direct cell-cell contact conditions. Most of these proteins are exosome cargos and are involved in cell motility and tissue development. These results indicate a dynamic interaction between MSC and GBM cells, favoring aggressive tumor cell traits through alternative and independent mechanisms. Overall, these findings indicate that MSC may exert pro-tumorigenic effects when in close contact with tumor cells, which must be carefully considered when employing MSC in targeted cell therapy protocols against cancer.

## INTRODUCTION

Glioblastoma (GBM) is a highly incident and fatal type of primary central nervous system tumor [1]. The typical fast and diffuse dissemination of GBM cells into the brain parenchyma is a critical factor that complicates tumor resection and facilitates tumor recurrence [2]. As a consequence, the prognosis of most GBM patients is very poor, with median survival rates of 12–15 months after maximal surgical resection followed by post-operative radiotherapy and/or adjuvant chemotherapy [3]. Understanding the mechanisms supporting the high proliferative and locally invasive behavior of GBM cells is therefore of great urgency.

Tumor growth and progression are long known to be affected by the tumor microenvironment. In addition to inflammatory cells, tumor-associated fibroblasts, endothelial cells, and pericytes, mesenchymal stem cells (MSC) are also actively attracted to primary tumor sites [4–6]. Within the tumor microenvironment, MSC may interact with tumor cells and secrete a large range of cytokines and growth factors that may contribute to tumor cell survival, growth, motility, and immune escape [7]. However, both pro-tumorigenic and tumor suppressor effects have been reported for MSC [8–13]. Due to their tumor-homing properties, MSC have also been genetically engineered and explored as cellular vehicles to deliver anti-cancer agents within tumor sites [14, 15].

MSC are known to secrete TGFB1 either as a soluble factor or via exosomes [16, 17]. This multifunctional cytokine of the transforming growth factor b family has been implicated in immunomodulation, proliferation, migration, and epithelial-mesenchymal transition (EMT) of tumor cells [18]. TGFB1 is also a well-known factor capable of increasing GBM cell proliferation and migration [19–22]. Given that autocrine TGFB1 signaling is essential to sustain stemness and high tumorigenicity of GBM-initiating cells [23], possible paracrine effects of cytokines released by resident MSC in the tumor stroma must be addressed.

In this study, consequences of the interaction between MSC and GBM cells to tumor development were evaluated by *in vitro* assays mimicking the tumor microenvironment, as well as *in vivo*. When in contact with the tumor cells, MSC significantly stimulated GBM cell proliferation, invasion and tumorigenesis. Notably, these oncogenic effects occurred independently of TGFB1 secretion by MSC, indicating alternative underlying mechanisms. Several proteins exclusively present in the secretome of MSC and GBM cell co-cultures were identified, revealing specific cell-to-cell signaling factors involved in their communication.

## RESULTS

### MSC-secreted TGFB1 significantly enhances GBM cell proliferation

**Figure 1:**
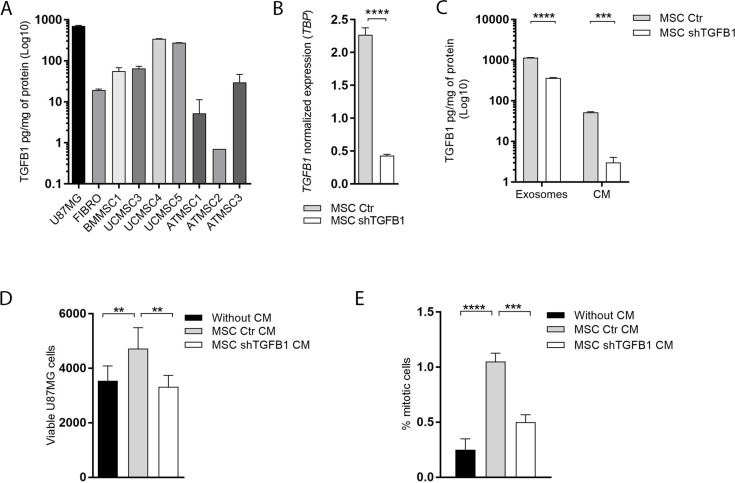
Effects of MSC-secreted TGFB1 on GBM cell proliferation (**A**) Basal TGFB1 protein levels secreted in conditioned medium (CM) by MSC derived from bone marrow (BMMSC1); umbilical cord (UCMSC3, UCMSC4 and UCMSC5) and adipose tissue (ATMSC1, ATMSC2 and ATMSC3). TGFB1 protein levels for U87MG and fibroblasts are shown for comparison. (**B**) Normalized *TGFB1* expression in MSC from umbilical cord (UCMSC4). **(C)** *TGFB1* knockdown significantly decreased TGFB1 protein levels in CM, and in exosomes of MSC. Total amount (**D**) and proliferation index (**E**) of viable U87MG cells cultured in the presence or absent of CM from transduced MSC. MSC Ctr. (MSC transduced with non-specific control plasmid); MSC shTGFB1 (MSC transduced with TGFB1 shRNA plasmid). Significance: ^*^p ≤ 0.05, ^**^p ≤ 0.01, ^****^p ≤ 0.0001.

An initial comparative analysis of TGFB1 production was carried out with seven human MSC samples harvested from different biological sources. Basal TGFB1 levels secreted in CM by MSC derived from bone marrow (BMMSC1); umbilical cord (UCMSC3, UCMSC4 and UCMSC5) and adipose tissue (ATMSC1, ATMSC2 and ATMSC3) are presented in Figure 1A. From this comparative quantification, the highest amount of TGFB1 was detected in the CM of UCMSC4 cultures, with cytokine levels comparable to the amount of TGFB1 secreted by GBM cells. The UCMSC4 sample was then used to generate MSC with stable *TGFB1* knockdown. *TGFB1* gene silencing was verified at the transcript level, reaching 81% reduction in expression (Figure 1B). Significant *TGFB1* knockdown was also confirmed at the protein level. Reductions of 94% and 69% were detected in TGFB1 content in MSC CM and in MSC-derived exosomes, respectively (Figure 1C). Respective reductions in TGFB1 protein levels were also confirmed in total protein extracts of MSC with a stable *TGFB1* knockdown (Supplementary Figure 1).

A functional indicator of the stable *TGFB1* knockdown in MSC was the significant increase in the amount of viable GBM cells detected after 72 h-incubation with CM from control MSC, but not with CM from TGFB1-deficient MSC (Figure 1D). In agreement with the literature [19–22], this result was correlated with a significant increase in GBM cell proliferation after incubation with CM from control MSC, which was not detected after incubation with CM from TGFB1-deficient MSC under the same experimental conditions (Figure 1E).

### GBM cell tumorigenicity is stimulated by contact with MSC independently of paracrine TGFB1

**Figure 2:**
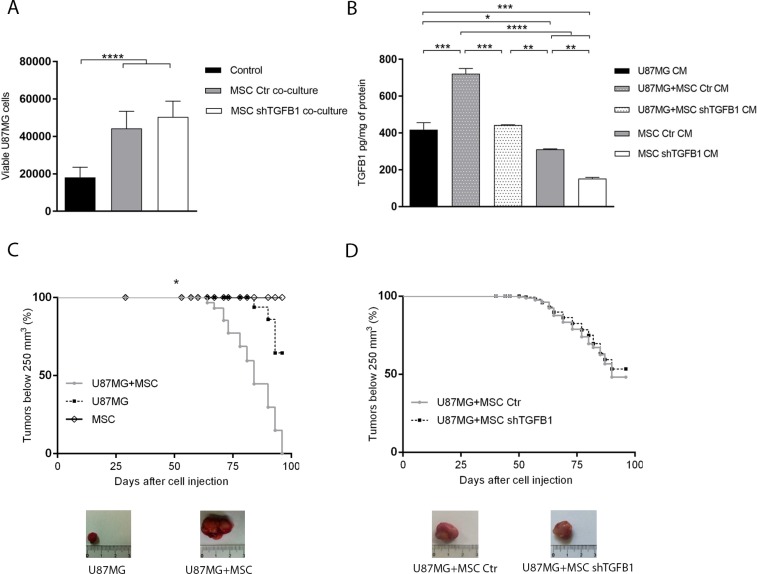
Effects of MSC on GBM cell tumorigenicity (**A**) Total amount of viable U87MG cells in single cultures or co-cultures with MSC allowing direct cell-cell contact. (**B**) TGFB1 protein levels in CM from U87MG and MSC single cultures, and in CM from U87MG–MSC co-cultures systems. (**C**) Kaplan–Meier plots of tumor growth after subcutaneous injection of MSC, U87MG cells, or U87MG cells in combination with MSC, in nude mice. Representative tumor images are shown. MSC injection did not generate tumors. (**D**) Kaplan–Meier plots of tumor growth after subcutaneous injection of U87MG cells with transduced MSC in nude mice. Representative tumor images are shown. MSC Ctr. (MSC transduced with non-specific control plasmid); MSC shTGFB1 (MSC transduced with TGFB1 shRNA plasmid). Significance: ^*^p ≤ 0.05, ^**^p ≤ 0.01, ^***^p ≤ 0.001, ^****^p ≤ 0.0001.

Co-cultivation of GBM cells with equal part of MSC, allowing direct cell-to-cell contact, significantly increased the amount of viable GBM cells after 72 h, when compared with standard GBM cell culture without MSC. Interestingly, this tumor cell population increment was detected in co-cultivation with either control MSC or TGFB1-deficient MSC (Figure 2A). Quantification of TGFB1 in the CM of these respective co-cultures confirmed normal TGFB1 secretion by control MSC, as well as impaired TGFB1 secretion by MSC subjected to *TGFB1* knockdown (Figure 2B).

Similarly, subcutaneous injection of GBM cells with an equal part of control MSC in BALBc/nude mice significantly increased tumor growth rate and final tumor volume, compared with injection of GBM cells alone. Under the same experimental conditions, no tumor formation was detected after injection of MSC only. Again, injections of GBM cells with either control MSC or TGFB1-deficient MSC generated tumors at similar rates, with no significant changes in final tumor size and weight (Figure 2C–2D and Supplementary Table 1).

**Figure 3:**
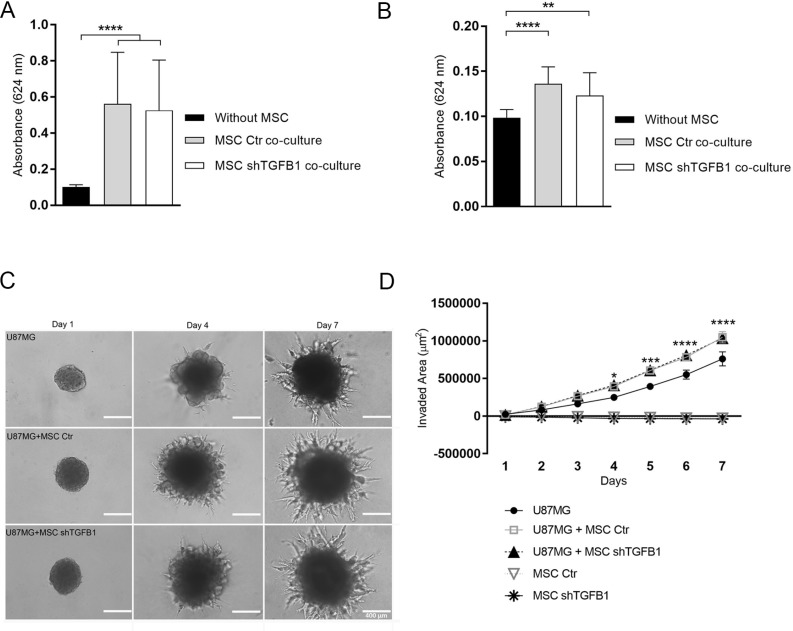
GBM cell migration and invasion capabilities are affected by MSC Tumor cells displayed significant chemoattraction to MSC, regardless the MSC-derived TGFB1 levels, showing increased migration (**A**) and invasion (**B**) compared with controls. (**C**) Presence of MSC enhanced U87MG 3-D cell invasion. GBM spheroids displayed significantly more protrusions in hydrogel matrix when co-cultured with MSC, regardless the MSC-derived TGFB1 levels. MSC did not show invasive properties in this assay. Photomicrographs at day 1, 4 and 7 for all groups. Scale bar: 400 µm. (**D**) Kinetics of 3-D cell invasion of U87MG, transduced MSC, and U87MG co-cultured with transduced MSC cells. MSC Ctr. (MSC transduced with non-specific control plasmid); MSC shTGFB1 (MSC transduced with TGFB1 shRNA plasmid). Significance: ^*^p ≤ 0.05, ^**^p ≤ 0.01, ^***^p ≤ 0.001, ^****^p ≤ 0.0001.

Similarly, co-cultivation of GBM cells with MSC, without direct cell-to-cell contact, elicited significant increases in both migration and invasion of tumor cells, compared with control conditions without MSC. In these assays, both control MSC and TGFB1-deficient MSC were equally able to stimulate GBM cell motility (Figure 3A–3B).

To better mimic tumor cell behavior *in vivo*, GBM cells were kept with or without direct contact with MSC and allowed to grow as 3D tumor spheroids embedded in a biological matrix. Tumor spheroids containing either control MSC or TGFB1-deficient MSC were highly enriched in spindle-like protrusions resulting from cells invading the hydrogel matrix (Figure 3C). Quantification of the total area of these protrusions over time revealed a significantly increased cell invaded area in tumor spheroids containing MSC, compared with tumor spheroids lacking MSC (Figure 3D). Again, such incremental effect on cell invasion was not affected by the level of MSC-secreted TGFB1.

**Figure 4:**
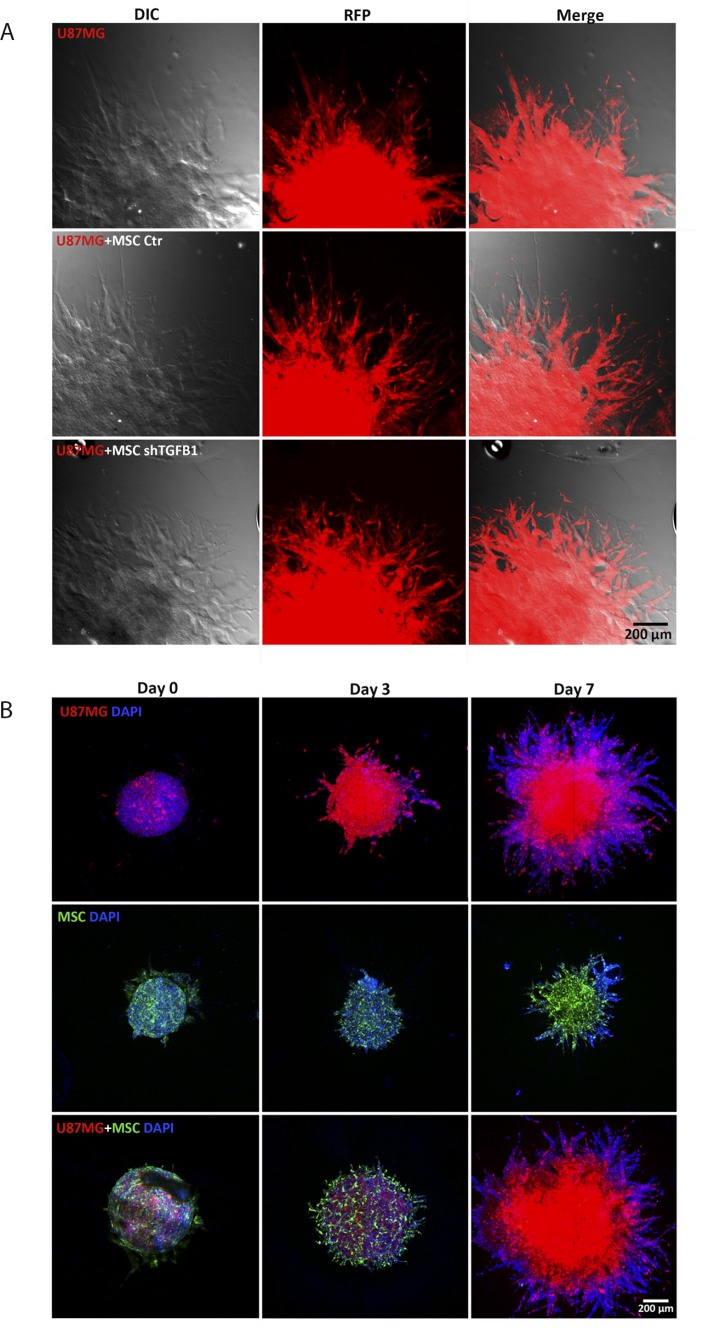
Detailed analysis of arboreal protrusions formed by cells invading the surrounding hydrogel matrix (**A**) Comparison between cell protrusions of spheroids from of U87MG cells alone and from U87MG cells with transduced MSC at day 7. Protrusions in the hydrogel matrix are mostly filled by red fluorescent U87MG cells. Scale bar: 400 µm. (**B**) 3D cell invasion experiment with U87MG cells co-cultivated with GFP+MSC demonstrating that MSC are visible in the tumor spheroids at day 0 and 3. At day 7, developed arboreal protrusions are predominantly filled by red fluorescent tumor cells. Spheroids comprised only by GFP+MSC or by FP635+U87MG cells are shown for comparison.

Since both MSC and tumor cells are capable of migrating, a more detailed analysis of the cell composition of the invading protrusions emanating from tumor spheroids was carried out. Confocal fluorescence microscopy images revealed that the highly invasive cells forming such protrusions were mainly GBM cells (U87MG) (Figure 4A). Since in this assay only tumor cells were fluorescent, further 3D invasion assays with tumor spheroids comprised by U87MG cells and GFP+ MSC were carried out. Although both GFP+ MSC and U87MG could be visualized in the initial tumor spheroids at balanced ratios, at day 7, the tumor spheroid core and invading protrusions were clearly dominated by U87MG cells, while GFP+ MSC could barely be detected. Spheroids comprised by GFP+ MSC only revealed a limited ability of these cells to invade the biological matrix (Figure 4B). These results confirmed prevalence of tumor cells in the invading protrusions of tumor/MSC spheroids and a stimulatory effect of MSC in U87MG cell invasion capacity.

### MSC–GBM cell contact elicit a unique secretome

**Figure 5:**
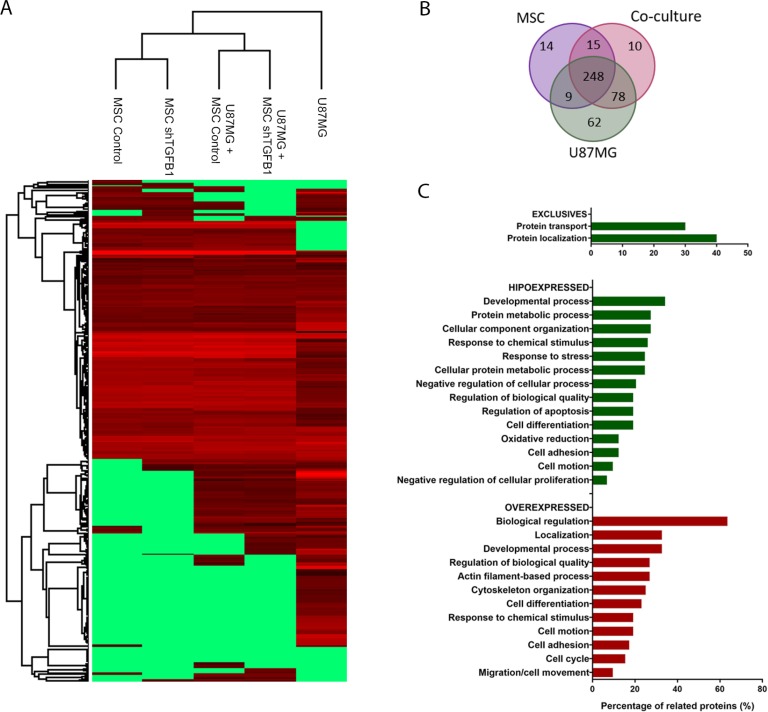
Secretome of MSC–GBM co-cultures (**A**) Cluster analysis of identified proteins present in CM of the following experimental groups: U87MG, U87MG+MSC shTGFB1, U87MG+MSC Ctr., MSC shTGFB1 and MSC Ctr. (**B**) Venn diagram displaying specific and common proteins identified in the cell culture conditions described above. (**C**) Functional annotation analysis of proteins found exclusively or differentially expressed in the CM of U87MG–MSC co-cultures.

Given that a direct cell-cell contact with MSC significantly increased tumorigenic properties of GBM cells despite TGFB1 silencing in the former cells, a comparative secretome analysis was carried out to identify alternative paracrine factors mediating the communication between MSC and GBM cells. As shown in the clustering dendrogram in Figure 5A, the proteomic profiles of CM from either MSC or TGFB1-deficient MSC single cultures were indeed highly similar to each other. Interestingly, the proteomic profiles of CM from MSC/U87MG and TGFB1-deficient MSC/U87MG co-cultures were also more similar to each other than with their single counterparts, suggesting particular changes in secretome due to direct cell-cell contact.

The Venn diagram in Figure 5B displays the total amount of proteins identified in the CM of either MSC cultures, U87MG culture, or MSC/U87MG co-cultures, depicting the amount of specific and common proteins among these groups. For this analysis, no distinction was made between MSC and TGFB1-deficient MSC, since both were equally able to affect tumorigenic properties of U87MG cells. Respective protein identities are presented in Supplementary Table 1. Notably, about 80% of the identified proteins are described in databases as constituents of extracellular exosomes. Of the 351 total proteins comprising the secretome of MSC/U87MG co-cultures, 10 proteins involved in cellular growth and proliferation were exclusively detected in this cell-cell contact condition, while other 126 proteins were found differentially expressed, as compared with the secretomes of MSC or U87MG cells, alone (Supplementary Tables 2–3).

A general functional classification of these differentially expressed proteins indicate roles in cell death and survival, cell morphology, cellular function and maintenance, cellular growth and proliferation, cellular movement and developmental processes (Figure 5C). This subset of proteins was particularly enriched in proteins involved in tumor metastasis and malignancy, such as proteins from the canonical actin cytoskeleton signaling, including several members of the RhoA/RhoGDI signaling pathway (ACTR2, EZR, MSM, PFN, GDI2).

## DISCUSSION

In the context of cancer, MSC are known to have tropism to tumor sites and, once incorporated in the tumor microenvironment, MSC may affect tumor development by releasing cytokines and growth factors, either directly or via exosomes. TGFB1 is one example of cytokine that is known to affect GBM cell proliferation. When produced by GBM cells, autocrine TGFB1 effects include stimulation of GBM stem cell tumorigenicity [20, 23]. Here, TGFB1 secreted by MSC was also capable of enhancing U87MG cell proliferation and viability, suggesting the importance of paracrine effects of this regulatory cytokine when produced by other cell types in the tumor microenvironment. Similarly, Sliwa *et al.* [24] demonstrated that TGFB1 derived from microglia stimulate the proliferation and invasion of glioma cells. Wesolowska *et al.* [25] also demonstrated that silencing *TGFB1 type II* receptor gene abolished GBM cell migration and invasion *in vitro* and decreased cell tumorigenicity in nude mice.

Despite the paracrine effects of MSC-derived TGFB1 observed in proliferation of tumor cells cultured with MSC conditioned media, a direct cell-cell contact between MSC and U87MG cells also stimulated tumorigenic properties of the latter and this effect was not dependent on TGFB1 levels produced by MSC. Under direct cell-cell contact in 3D conditions that better mimics what happens *in vivo*, tumor cells associated with MSC showed higher proliferative and invasive behavior. Notably, co-administration of U87MG cells with MSC significantly accelerated tumorigenesis in nude mice regardless the levels of TGFB1-secreted by MSC. These results reinforce the hypothesis that, as a tumor stromal component, MSC may promote GBM development given the effects observed in cell properties that are critical to GBM aggressiveness. High cellular proliferative rate and high capacity to invade the brain parenchyma, for instance, are key factors that limits complete surgical resection of GBM favoring tumor recurrence. These results are also in agreement with a recent study reporting that presence of MSC in the stroma of high grade gliomas correlates with poor patient survival [26].

The following comparative secretome study carried out indicates that interaction between U87MG cells and MSC was capable of modulating 136 proteins comprising a particular secretome profile correlated with the more aggressive tumor behavior observed *in vitro* and *in vivo*. Notably, although typical cellular communication involves soluble factors released in the extracellular medium, most of the proteins identified are extracellular exosome cargos, revealing an important contribution of exosomal delivery in the cell-cell communication between MSC and U87MG cells. It is important to notice, however, that these effects were detected under normal oxygen concentrations and that different cell-cell communication may occur under a hypoxic tumor microenvironment.

Paracrine effects of MSC exosomes have been associated with both tumor progression and suppression [27, 28]. In GBM cells, distinct effects caused by internalization of MSC-derived exosomes from various biological sources have been reported. While exosomes derived from AT-MSC stimulated cell proliferation of GBM, BM-MSC and UC-MSC exosomes inhibited proliferation and induced apoptosis [29]. Here, several proteins with known action on tumor cell invasion were found differentially expressed in the secretome of U87MG cells and MSC co-culture. Some of them, such as Profilin 2 (PFN2) and cortactin (CTTN), were exclusively found in the cell-cell contact condition.

PFN2 regulate actin polymerization in response to extracellular signals. A study by Kim *et al.* [30] indicated that the PFN2 affect the metastatic potential of colorectal cancer stem cells by regulating markers involved in the EMT (*E-cadherin* and *snail*) and pluripotency (*CD133*, *Sox2*, and *B-catenin*). In GBM tumors, a mesenchymal phenotype is correlated with increased radioresistance and poor patient survival [31]. CTTN is another cytoskeletal protein often overexpressed in invasive tumor cells. CTTN expression has been associated with tumor aggressiveness in leukemia [32], colorectal cancer [33], esophageal cancer [34], and hepatoma [35] among others. This protein modulates the Arp2/3 complex involved in cell migration and invasion, with a particular role in the formation of invadopodia [36]. CTTN was reported overexpressed in gliomas where its silencing significantly inhibit cell migration and invasion, as well as lamelipodia formation in tumor cells [37].

Ezrin (EZR) was another protein found overexpressed under U87MG cells and MSC direct contact. EZR has been associated with invasion of various types of cancer cells, including GBM [38–45]. In gliomas, EZR was also correlated with cell proliferation [46] and its expression was shown to be proportional to the degree of tumor malignancy [47, 48]. Another protein found overexpressed during cell-cell contact is ALCAM/CD166, a molecule highly expressed in GBM stem cells (CD133+). As for EZR, ALCAM expression was reported correlated with the histological grade of gliomas. Patient survival is significantly worse in tumors comprised by more than 60% of ALCAM+ cells [49]. In addition, GBM cells overexpressing a soluble isoform of ALCAM increased tumor cell invasion *in vitro* and were more tumorigenic *in vivo*. Interestingly, a study of TME in an animal model of GBM employed ALCAM expression, in addition to CD44 and CD91 expression, to characterize highly infiltrating GBM cells with mesenchymal characteristics [50].

In sum, these findings indicate that MSC can exert pro-tumorigenic effects on U87MG GBM cells by alternative and independent mechanisms, involving paracrine TGFB1 secretion and a direct cell-to-cell contact mechanism that is TGFB1-independent. The latter mechanism involves release of a particular set of exosomal proteins, some of which have been described to modulate tumor cell proliferation and invasion. The use of a single GBM cell line in the experimental model is a limitation of this study and further studies testing primary GBM cultures are desirable to gain mechanistic insight. Although only the proteome was evaluated in this study, exosomes are also known to carry mRNAs and microRNAs, whose contribution to the observed pro-oncogenic effects of MSC still need to be further addressed. Nevertheless, such undesirable oncogenic effects may impact cell therapy protocols employing MSC, particularly when planning the use of MSC as delivery systems to target cancer cells.

## MATERIALS AND METHODS

### Cell culture

U87MG human glioblastoma cells stably expressing far-red fluorescent protein (FP635, SHC013V, Sigma, St. Louis, MO, USA) were kindly provided by Dr. Vilma Regina Martins from the A.C. Camargo Cancer Center, São Paulo, Brasil. MSC derived from different human tissues were isolated and characterized as previously described [51]. Procedures were approved by the institutional review board (CEP-IB number 121/2011), and informed consent was obtained from all donors. All cells were cultivated with Dulbecco’s Modified Eagle Media (DMEM, Thermo Fisher Scientific, Waltham, MA, USA) supplemented with 10% Fetal Bovine Serum (FBS, Thermo Fisher Scientific), 100 U/mL Penicillin, 100 µg/mL Streptomycin and 250 ng/mL Fungizone^®^ (Thermo Fisher Scientific) at 37° C at 5% CO_2_ atmosphere. Exosomes were isolated from cell culture supernatants with ExoQuick^™^ (EXOQ5A-1, System Biosciences, Palo Alto, CA, USA), following the manufacturer’s instruction, and characterized by flow cytometry (FACS Aria II, Becton Dickinson, Franklin Lakes, NJ, USA) based on expression of CD81, CD9 and CD63.

### *TGFB1* knockdown in MSC

MSC cell with stable *TGFB1* knockdown (MSC shTGFB1) and non-specific control (MSC scrambled) were generated with TGFB1 silencing and control vectors, respectively (TG308855–HuSH shRNA Plasmid, OriGene Technologies, Rockville, MD, USA). Transduction and cell selection were performed according to the manufacture’s instruction. *TGFB1* knockdown efficiency was confirmed by Real Time qRT-PCR, performed as previously described [52], and by ELISA. For ELISA assays, MSC cell cultures at 60%–80% confluence had their medium discarded and cells were washed twice with PBS before fresh DMEM without FBS was added to the culture. Supernatants were harvested after 24 hours (h), centrifuged (250 g) to precipitate cells debris, and concentrated (1000 times) with Amicon Ultra-15 Centrifugal Filter Unit with 3 kDa cut-off (UFC900324 - Amicon Ultra-15; Millipore, Billerica, MA, USA). TGFB1 protein levels in cell culture supernatants, as well as in cell extracts and exosomes, were quantified using a Quantikine human TGFB1 immunoassay (SB100B - R&D Systems, Minneapolis, MN, USA). Total protein concentration was measured by Pierce BCA Protein Assay Kit (23225 - Thermo Fisher Scientific).

### Tumor cell proliferation in the presence of MSC conditioned medium

U87MG cells were previously synchronized and then seeded in DMEM with 0,5% FBS at a density of 1.25 10^4^/mL in 96-well plate for viable cell counting with Trypan Blue and in Nunc^™^ Lab-Tek^™^ II Chamber Slide^™^ System (154534 - Thermo Fisher Scientific) for mitotic index assay, as previously described [53]. Tumor cells were incubated for 72 h with or without conditioned medium (CM) from either MSC shTGFB1 or MSC scrambled cultures. First, volumes of CM MSC scrambled cultures were adjusted to a final TGFB1 concentration of 10 ng/mL in the assay, concentration that has been reported to exert sufficient influence on cell behavior [54, 55]. Then, corresponding amounts of total proteins in CM of MSC shTGFB1 and MSC scrambled cultures were used in the assays.

### Tumor cell quantification in co-culture assays

Co-culture assays were performed with direct cell-cell contact between tumor and MSC cells by plating 3,9 × 10^3^ cells/cm^2^ in DMEM supplemented with 0,5% FBS, in a U87MG:MSC proportion of 1:1. Co-culture assays were also carried out with indirect cell-cell contact through transwell chamber system (3422 - Corning™ Costar^™^ Transwell^™^ Permeable Supports, 0.4 µm pore size, Corning, NY, USA). In this system, tumor cells were cultured in inserts while MSC were cultured in bottom chambers at the same density/ratio. For both co-cultures, U87MG cultured without MSC were used as control for basal tumor cell proliferation. After 72 h of co-culture, viable fluorescent U87MG cells were counted in a Neubauer Chamber with Trypan Blue.

### Cell migration and invasion in co-culture assays

Tumor cell migration and invasion after 12 h of cell seeding in Boyden chambers were assayed as previously described [56]. MSC were seeded (3,9 × 10^3^ cells/cm^2^) in the bottom chambers without supplementation and allowed to attach overnight. The same amount of U87MG cells was seeded in inserts 24 h later, without supplementation. U87MG cells cultured without MSC were used as controls for basal tumor cell migration/invasion. U87MG cell invasion in the presence of absence of direct MSC contact was also assessed in a 3D tumor spheroid condition (3500-096-K - Cultrex^®^ 96 Well 3D Spheroid BME Cell Invasion Assay, Amsbio, Abingdon, UK). Cells were suspended in 50 µL of specialized Spheroid Formation ECM and plated into 96-well low-attachment round plate (7007–Corning) at a density of 6 × 10^4^ cells/mL to form spheres and embedded 24 h later in an invasion matrix. The spindle-like protrusions of invasive cells were visualized and quantified as previously described [53]. MSC cells stably expressing green fluorescent protein–GFP were used in the assay to distinguish MSC from RFP tumor cells within the spheres. Tumor spheres were fixed in 3.7% formaldehyde solution for 40 min, permeabilized with 0.5% Triton X-100 for 30 minutes (min), incubated with DAPI for 45 min, and mounted in Vectashield^®^ before visualization in a Confocal microscope (Zeiss LSM 780-NLO Confocal Microscope).

### Tumorigenesis assay

Tumor development in 9-week-old BALB/c nude mice was assessed as previously described [56]. Cell suspensions containing 100 µL of either MSC, U87MG or both (10^6^ cells of each, 1:1 ratio) were administrated subcutaneously in the flank using an insulin syringe (Becton Dickinson), and tumor growth was monitored weekly. The study was approved by the ethics committee for animal research of the University of São Paulo (CEUA protocol no. 132/2011).

### Secretome profiling

Proteins in the supernatant of U87MG and MSC cell single cultures, as well as in U87MG–MSC direct co-cultures were determined by liquid chromatography coupled to tandem mass spectrometry (LC-MS/MS). A detailed description of the methodology is provided in the supplementary material and methods. The mass spectrometry proteomics data has been deposited to the ProteomeXchange Consortium via the PRoteomics IDEntifications (PRIDE) [57] partner repository with the dataset identifier PXD008035.

### Statistical analysis

All experiments were performed in triplicate and three independent experiments were carried out. The results were analyzed comparatively between the control and experimental groups. Data were expressed as mean ± SD and analyzed by Student *t*-test or analysis of variance (ANOVA) complemented by Tukey or Bonferroni post hoc test. The significance level of *p* < 0.05 was adopted for all experiments.

## SUPPLEMENTARY MATERIALS FIGURE AND TABLES






