# Supplementary material for: Mesenchymal stem cells enhance tumorigenic properties of human glioblastoma through independent cell-cell communication mechanisms
Source: Oncotarget. 2018 May 15;9(37):24766–77. doi: 10.18632/oncotarget.25346 (PMC5973871; doi:10.18632/oncotarget.25346)
Supplement: Supplementary file 1 [file oncotarget-09-24766-s001.pdf]

Mesenchymal stem cells enhance tumorigenic properties of human glioblastoma through independent cell-cell communication mechanisms

SUPPLEMENTARY MATERIALS

Real –time PCR

The primer sequences were as follows (5′ to 3′): *TGFB1* Forward: AAGGACCTCGGCTGGAAGTG, *TGFB1* Reverse: CCCGGGTTATGCTGGTTGTA; *TBP* Forward: GAGCTGTGATGTGAAGTTTCC, *TBP* Reverse: TCTGGGTTTGATCATTCTGTAG.

Proteomics

Protein digestion

For protein digestion, 50 µg of total protein from CM co-cultures and respective controls was diluted in 50 mM ammonium bicarbonate to a final volume of 60 µL. Then, protein samples were denatured with 0.2% (wt:vol) RapiGest™ SF Surfactant (Waters) at 80° C for 15 min, reduced with 2.5 µL of 100 mM dithiothreitol at 60° C for 30 min, alkylated with 2.5 µL of 300 mM iodoacetamide at room temperature for 30 min, and enzymatically digested at 37° C overnight with trypsin at a 1:50 (wt/wt) enzyme:protein ratio. After, hydrolyze the RapiGest with 10 µL of 5% trifluoroacetic acid (TFA) and samples were incubated at 37° C for 90 min. Centrifuged samples at 16,000 × g and 6° C for 30 min and 16 µL of the internal standard (yeast alcohol dehydrogenase at 1 pmol/µL) were added.

Processing samples and analyzing protein profile by liquid chromatography coupled to tandem mass spectrometry (LC-MS/MS)

Samples were analyzed by nanoACQUITY system (Waters) which contains two binary nanoHPLC pumps. Peptides were desalted and concentrated in a Symmetry C18 column (180 µm × 20 mm, 5 µm; Waters) with 0.1% TFA (15 µL/min for 5 min), and directed to HSS C18 analytical column (75 µm × 150 mm, 1.7 µm; Waters) where they were separated by a gradient elution with a mixture of 2 % DMSO in 0.1% formic acid in water and 5% DMSO in 0.1% formic acid in acetonitrile which proportion was increased from 2 to 45% in 90 minutes at 0.4 µL/min.

Protein identification and database analysis

Acquisition of four to nine technical replicates of samples were made. The tandem mass spectrometer Q-Exactive type (Thermo Scientific) was calibrated in the positive mode at the beginning of each analytical sequence. Analytical sequence started after the acquisition of human cell line extract Mino (new mantle cell lymphoma) for quality control of the number of identified proteins [1] the t(11;14. The acquired data were processed by MaxQuant programs [2] specifically designed for high-resolution and quantitative data. Protein identification was performed by Andromeda search tool using the database of the human proteome UniProtKB (Swissprot june 2015). Following criteria were applied to identification: allowed maximum of two incomplete cleavages by trypsin, fixed modification by carbamidometilation of cysteines and variable changes by N-terminal portion acetylation and methionine oxidation. Quantification was based on a modified intensity based absolute quantification label-free strategy [3] in which P00330 was used as internal normalizer. Cross-validation of protein identification through the Trans-Proteomic Pipeline platform was performed (TPP) using the X! Tandem as search tool and applying the same parameters used in the analysis by Andromeda/MaxQuant.

Statistical analysis

Student *t*-test was performed and $p < 0.05$ were considered significant at Perseus software. The proteins identified in MaxQuant platform were considered for analysis only if they could obtain probabilities above 95% in Protein Prophet system module X!Tandem/TPP.

Functional analysis systems biology

Analyzes were performed using the Ingenuity Pathway Analysis software (IPA). The reference database used was the Ingenuity Knowledge Base (Genes) including only direct relations considering molecules and relationships in humans and experimentally observed.

Complementary analysis of Functional Annotation was carried out with DAVID Bioinformatics Resources 6.8.

SUPPLEMENTARY REFERENCES

1. Lai R, McDonnell TJ, O'Connor SL, Medeiros LJ, Oudat R, Keating M, Morgan MB, Curiel TJ, Ford RJ. Establishment and characterization of a new mantle cell lymphoma cell line, Mino. Leuk Res. 2002; 26:849–55.
2. Cox J, Mann M. MaxQuant enables high peptide identification rates, individualized p.p.b.-range mass accuracies and proteome-wide protein quantification. Nat Biotechnol. 2008; 26:1367–72. https://doi.org/10.1038/nbt.1511.
3. Schwanhäusser B, Busse D, Li N, Dittmar G, Schuchhardt J, Wolf J, Chen W, Selbach M. Global quantification of mammalian gene expression control. Nature. 2011; 473: 337–42. https://doi.org/10.1038/nature10098.

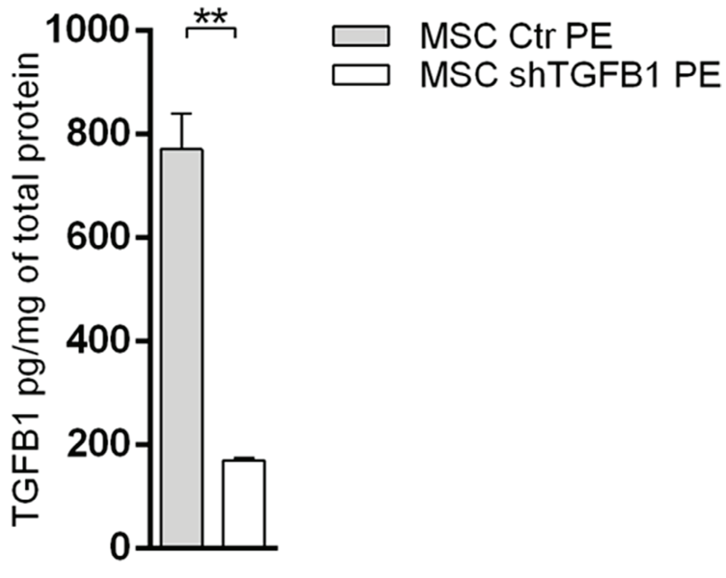

Supplementary Figure 1: Confirmation of stable *TGFB1* knockdown in MSC by ELISA assays. TGFB1 protein levels in total protein extracts (PE) of MSC transduced with shRNA targeting *TGFB1* were significantly lower than in PE of MSC transduced with non-specific control plasmid.

Supplementary Table 1: *In vivo* tumorigenesis

Group	Animal (*n*)	Tumor (*n*)	Tumor weight (g)-mean (SD)
U87MG	5	3	0.47 (0.28)
U87MG + MSC	5	3	1.74 (0.04)
U87MG + MSC Ctr	12	9	0.81 (0.55)
U87MG + MSC shTGFB1	12	6	0.79 (0.56)

Number of animals inoculated, tumors detected and respective weight are shown for all experimental groups.

Supplementary Table 2: Proteins identified by *LC-MS/MS* in conditioned medium for all experimental groups: U87MG single culture, MSC single culture, and U87MG + MSC co-culture. See Supplementary_Table_2

Supplementary Table 3: Exclusive and differentially expressed (over or hipoexpressed) proteins identified by *LC-MS/MS* in conditioned medium from U87MG + MSC co-culture, as compared with controls (MSC and U87MG single cultures). See Supplementary_Table_3
