# Supplementary material for: Mesenchymal stem cells enhance tumorigenic properties of human glioblastoma through independent cell-cell communication mechanisms
Source: Oncotarget. 2018 May 15;9(37):24766–77. doi: 10.18632/oncotarget.25346 (PMC5973871; doi:10.18632/oncotarget.25346)
Supplement: Supplementary file 2 [file oncotarget-09-24766-s002.docx]

Supplementary Table 2: Proteins identified by *LC-MS/MS* in conditioned medium for all experimental groups: U87MG single culture, MSC single culture, and U87MG + MSC co-culture.

| **Proteins common to the three groups (n=248)** | **UniProt** | **Protein name** |
| --- | --- | --- |
|  | P46940 | IQ motif containing GTPase activating protein 1 |
|  | P07437 | tubulin, beta 5; tubulin, beta pseudogene 2; tubulin, beta pseudogene 1 |
|  | P17655 | calpain 2, (m/II) large subunit |
|  | P78417 | glutathione S-transferase omega 1 |
|  | P27816 | microtubule-associated protein 4 |
|  | O76061 | stanniocalcin 2 |
|  | P37802 | transgelin 2 |
|  | P23528 | cofilin 1 (non-muscle) |
|  | O14786 | neuropilin 1 |
|  | P61769 | beta-2-microglobulin |
|  | P07737 | profilin 1 |
|  | P11142 | heat shock 70kDa protein 8 |
|  | P98160 | heparan sulfate proteoglycan 2 |
|  | P28066 | proteasome (prosome, macropain) subunit, alpha type, 5 |
|  | Q8NBS9 | thioredoxin domain containing 5 (endoplasmic reticulum); muted homolog (mouse) |
|  | P41250 | glycyl-tRNA synthetase |
|  | Q92520 | family with sequence similarity 3, member C |
|  | O75369 | filamin B, beta (actin binding protein 278) |
|  | P14314 | protein kinase C substrate 80K-H |
|  | P62258 | 14-3-3 protein epsilon |
|  | P00558 | phosphoglycerate kinase 1 |
|  | P07237 | prolyl 4-hydroxylase, beta polypeptide |
|  | P13797 | plastin 3 (T isoform) |
|  | Q10471 | UDP-N-acetyl-alpha-D-galactosamine:polypeptide N-acetylgalactosaminyltransferase 2 (GalNAc-T2) |
|  | Q02818 | nucleobindin 1 |
|  | Q99536 | vesicle amine transport protein 1 homolog (T. californica) |
|  | P18206 | vinculin |
|  | P52565 | Rho GDP dissociation inhibitor (GDI) alpha |
|  | P09871 | complement component 1, s subcomponent |
|  | P16152 | carbonyl reductase 1 |
|  | P13667 | protein disulfide isomerase family A, member 4 |
|  | P27348 | tyrosine 3-monooxygenase/tryptophan 5-monooxygenase activation protein, theta polypeptide |
|  | P00491 | nucleoside phosphorylase |
|  | Q58FF8 | heat shock protein 90kDa alpha (cytosolic), class B member 2 (pseudogene) |
|  | P14625 | heat shock protein 90kDa beta (Grp94), member 1 |
|  | P09486 | secreted protein, acidic, cysteine-rich (osteonectin) |
|  | O75083 | WD repeat domain 1 |
|  | P09382 | lectin, galactoside-binding, soluble, 1 |
|  | P11021 | hypothetical gene supported by AF216292; NM_005347; heat shock 70kDa protein 5 (glucose-regulated protein, 78kDa) |
|  | P63104 | tyrosine 3-monooxygenase/tryptophan 5-monooxygenase activation protein, zeta polypeptide |
|  | Q13219 | PAPPA antisense RNA (non-protein coding); pregnancy-associated plasma protein A, pappalysin 1 |
|  | Q5VTE0 | eukaryotic translation elongation factor 1 alpha-like 7; alpha-like 3; alpha 1 |
|  | Q02809 | procollagen-lysine 1, 2-oxoglutarate 5-dioxygenase 1 |
|  | Q9H299 | SH3 domain binding glutamic acid-rich protein like 3 |
|  | P30101 | protein disulfide isomerase family A, member 3 |
|  | P22692 | insulin-like growth factor binding protein 4 |
|  | P00338 | lactate dehydrogenase A |
|  | P61088 | ubiquitin-conjugating enzyme E2N (UBC13 homolog, yeast) |
|  | P07108 | diazepam binding inhibitor (GABA receptor modulator, acyl-Coenzyme A binding protein) |
|  | Q15582 | transforming growth factor, beta-induced, 68kDa |
|  | P62987 | ubiquitin A-52 residue ribosomal protein fusion product 1 |
|  | Q15084 | protein disulfide isomerase family A, member 6 |
|  | P06576 | ATP synthase, H+ transporting, mitochondrial F1 complex, beta polypeptide |
|  | P06396 | gelsolin (amyloidosis, Finnish type) |
|  | O75874 | isocitrate dehydrogenase 1 (NADP+), soluble |
|  | P23284 | peptidylprolyl isomerase B (cyclophilin B) |
|  | P14618 | Pyruvate kinase, isozymes M1/M2 (Pyruvate kinase muscle isozyme) (Cytosolic thyroid hormone-binding protein) |
|  | P07602 | prosaposin |
|  | P23381 | tryptophanyl-tRNA synthetase |
|  | P01033 | TIMP metallopeptidase inhibitor 1 |
|  | P17174 | glutamic-oxaloacetic transaminase 1, soluble (aspartate aminotransferase 1) |
|  | Q07021 | complement component 1, q subcomponent binding protein |
|  | P60709 | actin, beta |
|  | P31946 | tyrosine 3-monooxygenase/tryptophan 5-monooxygenase activation protein, beta polypeptide |
|  | P29401 | transketolase |
|  | P61604 | heat shock 10kDa protein 1 (chaperonin 10) |
|  | P16035 | TIMP metallopeptidase inhibitor 2 |
|  | P13489 | ribonuclease/angiogenin inhibitor 1 |
|  | P62328 | thymosin-like 2 (pseudogene); thymosin-like 1 (pseudogene); thymosin beta 4, X-linked |
|  | P05121 | serpin peptidase inhibitor, clade E (nexin, plasminogen activator inhibitor type 1), member 1 |
|  | P31949 | S100 calcium binding protein A11; S100 calcium binding protein A11 pseudogene |
|  | P01024 | similar to Complement C3 precursor; complement component 3; hypothetical protein LOC100133511 |
|  | P09651 | heterogeneous nuclear ribonucleoprotein A1-like 3; A1; A1 pseudogene 2; A1 pseudogene |
|  | Q06830 | peroxiredoxin 1 |
|  | Q6EMK4 | vasorin |
|  | P00736 | complement component 1, r subcomponent |
|  | P40925 | malate dehydrogenase 1, NAD (soluble) |
|  | P40926 | malate dehydrogenase 2, NAD (mitochondrial) |
|  | O75326 | semaphorin 7A, GPI membrane anchor (John Milton Hagen blood group) |
|  | P60903 | S100 calcium binding protein A10 |
|  | Q15019 | septin 2 |
|  | P21333 | filamin A, alpha (actin binding protein 280) |
|  | P08123 | collagen, type I, alpha 2 |
|  | O94985 | calsyntenin 1 |
|  | Q13813 | spectrin, alpha, non-erythrocytic 1 (alpha-fodrin) |
|  | Q9BRK5 | stromal cell derived factor 4 |
|  | P60900 | proteasome (prosome, macropain) subunit, alpha type, 6 |
|  | P24534 | eukaryotic translation elongation factor 1 beta 2; 1 beta 2-like |
|  | P04792 | heat shock 27kDa protein-like 2 pseudogene; heat shock 27kDa protein 1 |
|  | P13611 | versican |
|  | P01034 | cystatin C |
|  | P25788 | proteasome (prosome, macropain) subunit, alpha type, 3 |
|  | P25786 | proteasome (prosome, macropain) subunit, alpha type, 1 |
|  | P25787 | proteasome (prosome, macropain) subunit, alpha type, 2 |
|  | P00441 | superoxide dismutase 1, soluble |
|  | P27797 | calreticulin |
|  | P30086 | phosphatidylethanolamine binding protein 1 |
|  | P30085 | cytidine monophosphate (UMP-CMP) kinase 1, cytosolic |
|  | P61981 | tyrosine 3-monooxygenase/tryptophan 5-monooxygenase activation protein, gamma polypeptide |
|  | Q01995 | transgelin |
|  | O60568 | procollagen-lysine, 2-oxoglutarate 5-dioxygenase 3 |
|  | Q16610 | extracellular matrix protein 1 |
|  | P07900 | heat shock protein 90kDa alpha (cytosolic), class A member 2; class A member 1 |
|  | P08670 | vimentin |
|  | Q09666 | AHNAK nucleoprotein |
|  | P48643 | chaperonin containing TCP1, subunit 5 (epsilon) |
|  | P12110 | collagen, type VI, alpha 2 |
|  | P26641 | eukaryotic translation elongation factor 1 gamma |
|  | O43854 | EGF-like repeats and discoidin I-like domains 3 |
|  | P13639 | eukaryotic translation elongation factor 2 |
|  | Q14697 | glucosidase, alpha; neutral AB |
|  | P12109 | collagen, type VI, alpha 1 |
|  | Q9UNN8 | protein C receptor, endothelial (EPCR) |
|  | O43852 | calumenin |
|  | Q16270 | insulin-like growth factor binding protein 7 |
|  | Q01082 | spectrin, beta, non-erythrocytic 1 |
|  | P04406 | glyceraldehyde-3-phosphate dehydrogenase-like 6; glyceraldehyde-3-phosphate dehydrogenase |
|  | P09936 | ubiquitin carboxyl-terminal esterase L1 (ubiquitin thiolesterase) |
|  | Q9ULV4 | coronin, actin binding protein, 1C |
|  | P19022 | cadherin 2, type 1, N-cadherin (neuronal) |
|  | P19021 | peptidylglycine alpha-amidating monooxygenase |
|  | P08865 | ribosomal protein SA pseudogene 9; 8;58;19;18;15;61;29;12 |
|  | P55786 | hypothetical protein FLJ11822; aminopeptidase puromycin sensitive |
|  | P53396 | ATP citrate lyase |
|  | O43707 | actinin, alpha 4 |
|  | P61978 | heterogeneous nuclear ribonucleoprotein K; similar to heterogeneous nuclear ribonucleoprotein K |
|  | Q9UBP4 | dickkopf homolog 3 (Xenopus laevis) |
|  | Q99497 | Parkinson disease (autosomal recessive, early onset) 7 |
|  | P04080 | cystatin B (stefin B) |
|  | P68036 | ubiquitin-conjugating enzyme E2L 3 |
|  | P04083 | annexin A1 |
|  | Q9Y490 | talin 1 |
|  | P12111 | collagen, type VI, alpha 3 |
|  | P18669 | phosphoglycerate mutase 1 (brain) |
|  | P08572 | collagen, type IV, alpha 2 |
|  | P22626 | heterogeneous nuclear ribonucleoprotein A2/B1 |
|  | P06703 | S100 calcium binding protein A6 |
|  | Q15113 | procollagen C-endopeptidase enhancer |
|  | Q15293 | reticulocalbin 1, EF-hand calcium binding domain |
|  | Q9Y240 | C-type lectin domain family 11, member A |
|  | P60842 | similar to eukaryotic translation initiation factor 4A; small nucleolar RNA, H/ACA box 67 |
|  | Q15149 | Plectin 1 (PLTN) (PCN) (Hemidesmosomal protein 1) (HD1);intermediate filament binding protein 500kDa |
|  | P02545 | lamin A/C |
|  | O00299 | chloride intracellular channel 1 |
|  | P55290 | cadherin 13, H-cadherin (heart) |
|  | P08238 | heat shock protein 90kDa alpha (cytosolic), class B member 1 |
|  | P09211 | glutathione S-transferase pi 1 |
|  | P10599 | thioredoxin |
|  | P24592 | insulin-like growth factor binding protein 6 |
|  | P67936 | tropomyosin 4 |
|  | P63241 | eukaryotic translation initiation factor 5A; eukaryotic translation initiation factor 5A-like 1 |
|  | Q15262 | protein tyrosine phosphatase, receptor type, K |
|  | P34932 | heat shock 70kDa protein 4 |
|  | P80723 | brain abundant, membrane attached signal protein 1 |
|  | Q14974 | karyopherin (importin) beta 1 |
|  | O00469 | procollagen-lysine, 2-oxoglutarate 5-dioxygenase 2 |
|  | P04075 | aldolase A, fructose-bisphosphate |
|  | Q15121 | phosphoprotein enriched in astrocytes 15 |
|  | P12814 | actinin, alpha 1 |
|  | P30041 | peroxiredoxin 6 |
|  | P19105 | myosin, light chain 12A, regulatory, non-sarcomeric |
|  | P52907 | capping protein (actin filament) muscle Z-line, alpha 1 |
|  | P30044 | peroxiredoxin 5 |
|  | P22392 | non-metastatic cells 1, protein (NM23A); NME1-NME2 readthrough transcript; non-metastatic cells 2, protein (NM23B) |
|  | P30046 | D-dopachrome tautomerase |
|  | P68032 | actin, alpha, cardiac muscle 1 |
|  | Q00610 | clathrin, heavy chain (Hc) |
|  | P60174 | TPI1 pseudogene; triosephosphate isomerase 1 |
|  | Q9Y617 | chromosome 8 open reading frame 62; phosphoserine aminotransferase 1 |
|  | Q9BRA2 | thioredoxin domain containing 17 |
|  | P05388 | ribosomal protein, large, P0 pseudogene 2;3;6 |
|  | O15143 | Actin-related protein 2/3 complex subunit 1B (ARP2/3 complex 41 kDa subunit) (p41-ARC) |
|  | Q12841 | follistatin-like 1 |
|  | P35237 | serpin peptidase inhibitor, clade B (ovalbumin), member 6 |
|  | P07195 | lactate dehydrogenase B |
|  | P50454 | serpin peptidase inhibitor, clade H (heat shock protein 47), member 1, (collagen binding protein 1) |
|  | P05067 | amyloid beta (A4) precursor protein |
|  | Q9UI42 | carboxypeptidase A4 |
|  | P09603 | colony stimulating factor 1 (macrophage) |
|  | P50395 | GDP dissociation inhibitor 2 |
|  | Q16658 | fascin homolog 1, actin-bundling protein (Strongylocentrotus purpuratus) |
|  | P36222 | chitinase 3-like 1 (cartilage glycoprotein-39) |
|  | P26022 | pentraxin-related gene, rapidly induced by IL-1 beta |
|  | P08253 | matrix metallopeptidase 2 (gelatinase A, 72kDa gelatinase, 72kDa type IV collagenase) |
|  | O60664 | mannose-6-phosphate receptor binding protein 1 |
|  | Q6IBS0 | twinfilin, actin-binding protein, homolog 2 (Drosophila) |
|  | Q08380 | lectin, galactoside-binding, soluble, 3 binding protein |
|  | P07093 | serpin peptidase inhibitor, clade E (nexin, plasminogen activator inhibitor type 1), member 2 |
|  | P49720 | proteasome (prosome, macropain) subunit, beta type, 3 |
|  | P07355 | annexin A2 pseudogene 3; annexin A2; annexin A2 pseudogene 1 |
|  | P20908 | collagen, type V, alpha 1 |
|  | P15291 | UDP-Gal:betaGlcNAc beta 1,4- galactosyltransferase, polypeptide 1 |
|  | P06733 | enolase 1, (alpha) |
|  | P14174 | macrophage migration inhibitory factor (glycosylation-inhibiting factor) |
|  | P68371 | tubulin, beta 2C |
|  | O00391 | quiescin Q6 sulfhydryl oxidase 1 |
|  | P55058 | phospholipid transfer protein |
|  | P16930 | fumarylacetoacetate hydrolase (fumarylacetoacetase) |
|  | P78371 | chaperonin containing TCP1, subunit 2 (beta) |
|  | P22314 | ubiquitin-like modifier activating enzyme 1 |
|  | P26038 | moesin |
|  | P68363 | hypothetical gene supported by AF081484; NM_006082; tubulin, alpha 1b |
|  | P06744 | glucose phosphate isomerase |
|  | Q9UHI8 | ADAM metallopeptidase with thrombospondin type 1 motif, 1 |
|  | P60981 | destrin (actin depolymerizing factor) |
|  | P35579 | myosin, heavy chain 9, non-muscle |
|  | P09972 | aldolase C, fructose-bisphosphate |
|  | P54727 | RAD23 homolog B (S. cerevisiae) |
|  | P13693 | tumor protein, translationally-controlled 1 |
|  | P06753 | tropomyosin 3 |
|  | O14818 | proteasome (prosome, macropain) subunit, alpha type, 7 |
|  | P10809 | heat shock 60kDa protein 1 (chaperonin) pseudogene 5; 6 ; 1 ;4 |
|  | P52209 | phosphogluconate dehydrogenase |
|  | P10909 | clusterin |
|  | Q16881 | thioredoxin reductase 1; hypothetical LOC100130902 |
|  | P15121 | aldo-keto reductase family 1, member B1 (aldose reductase) |
|  | P61158 | ARP3 actin-related protein 3 homolog (yeast) |
|  | P46821 | microtubule-associated protein 1B |
|  | P35442 | thrombospondin 2 |
|  | P02751 | fibronectin 1 |
|  | Q14019 | coactosin-like 1 (Dictyostelium) |
|  | P07711 | cathepsin L1 |
|  | P61916 | Niemann-Pick disease, type C2 |
|  | Q6YHK3 | CD109 molecule |
|  | P17936 | insulin-like growth factor binding protein 3 |
|  | P21291 | cysteine and glycine-rich protein 1 |
|  | P62158 | calmodulin 3 ; calmodulin 2; calmodulin 1 (phosphorylase kinase, delta) |
|  | Q16555 | dihydropyrimidinase-like 2 |
|  | P61160 | ARP2 actin-related protein 2 homolog (yeast) |
|  | P11047 | laminin, gamma 1 (formerly LAMB2) |
|  | P07996 | thrombospondin 1 |
|  | Q14118 | dystroglycan 1 (dystrophin-associated glycoprotein 1) |
|  | Q01518 | CAP, adenylate cyclase-associated protein 1 (yeast) |
|  | P60660 | myosin, light chain 6, alkali, smooth muscle and non-muscle |
|  | P07858 | cathepsin B |
|  | P35555 | fibrillin 1 |
|  | Q14315 | filamin C, gamma (actin binding protein 280) |
|  | P62937 | similar to TRIMCyp; peptidylprolyl isomerase A (cyclophilin A); peptidylprolyl isomerase A (cyclophilin A)-like 3 |
|  | P35754 | glutaredoxin (thioltransferase) |
|  | P51884 | lumican |
|  | Q08629 | sparc/osteonectin, cwcv and kazal-like domains proteoglycan (testican) 1 |
|  | P07339 | cathepsin D |
|  | Q13740 | hypothetical protein LOC100133690; activated leukocyte cell adhesion molecule |
|  | P28838 | leucine aminopeptidase 3 |
|  | P16070 | CD44 molecule (Indian blood group) |
|  | Q08431 | milk fat globule-EGF factor 8 protein |
|  | P00330 | ADH1, Alcohol dehydrogenase 1 |
|  | P0DMV9 | HSPA1B, Heat shock 70 kDa protein 1B |
|  |  |  |
| **Proteins common between co-culture and MSC (n=15)** | **UniProt** | **Protein name** |
|  | P05997 | collagen, type V, alpha 2 |
|  | Q96D15 | reticulocalbin 3, EF-hand calcium binding domain |
|  | O00468 | agrin |
|  | O75368 | SH3 domain binding glutamic acid-rich protein like |
|  | P55287 | cadherin 11, type 2, OB-cadherin (osteoblast) |
|  | P02462 | collagen, type IV, alpha 1 |
|  | P07585 | decorin |
|  | P00750 | plasminogen activator, tissue |
|  | P29966 | myristoylated alanine-rich protein kinase C substrate |
|  | P24593 | insulin-like growth factor binding protein 5 |
|  | P21810 | biglycan |
|  | P55072 | valosin-containing protein |
|  | Q9Y4K0 | lysyl oxidase-like 2 |
|  | Q9BUD6 | spondin 2, extracellular matrix protein |
|  | P03956 | matrix metallopeptidase 1 (interstitial collagenase) |
|  |  |  |
| **Proteins common between U87MG and MSC (n=9)** | **UniProt** | **Protein name** |
|  | Q13443 | ADAM metallopeptidase domain 9 (meltrin gamma) |
|  | O75347 | tubulin folding cofactor A |
|  | Q14112 | nidogen 2 (osteonidogen) |
|  | O75531 | similar to barrier-to-autointegration factor; barrier to autointegration factor 1 |
|  | P62857 | ribosomal protein S28 pseudogene 6; ribosomal protein S28 pseudogene 9; ribosomal protein S28 |
|  | P14550 | aldo-keto reductase family 1, member A1 (aldehyde reductase) |
|  | P80303 | nucleobindin 2 |
|  | P32119 | peroxiredoxin 2 |
|  | P30050 | ribosomal protein L12 pseudogene 2;32;35;19;6;14 |
|  |  |  |
| **Proteins common between co-culture and U87MG (n=78)** | **UniProt** | **Protein name** |
|  | Q16531 | damage-specific DNA binding protein 1, 127kDa |
|  | P33316 | deoxyuridine triphosphatase |
|  | Q13765 | nascent polypeptide-associated complex alpha subunit |
|  | P00367 | glutamate dehydrogenase 1 |
|  | Q13200 | proteasome (prosome, macropain) 26S subunit, non-ATPase, 2 |
|  | P01892 | major histocompatibility complex, class I, A |
|  | Q16769 | glutaminyl-peptide cyclotransferase |
|  | P29692 | eukaryotic translation elongation factor 1 delta (guanine nucleotide exchange protein) |
|  | P31948 | stress-induced-phosphoprotein 1 |
|  | P30153 | protein phosphatase 2 (formerly 2A), regulatory subunit A, alpha isoform |
|  | P07686 | hexosaminidase B (beta polypeptide) |
|  | Q92820 | gamma-glutamyl hydrolase (conjugase, folylpolygammaglutamyl hydrolase) |
|  | P23526 | adenosylhomocysteinase |
|  | P26006 | integrin, alpha 3 (antigen CD49C, alpha 3 subunit of VLA-3 receptor) |
|  | Q13822 | ectonucleotide pyrophosphatase/phosphodiesterase 2 |
|  | P15311 | hypothetical protein LOC100129652; ezrin |
|  | Q9BWS9 | chitinase domain containing 1 |
|  | P00568 | adenylate kinase 1 |
|  | P62826 | RAN, member RAS oncogene family |
|  | P28799 | granulin |
|  | Q16543 | cell division cycle 37 homolog (S. cerevisiae) |
|  | P78539 | sushi-repeat-containing protein, X-linked |
|  | Q01105 | SET nuclear oncogene; similar to SET translocation |
|  | P30530 | AXL receptor tyrosine kinase |
|  | P05556 | integrin, beta 1 (fibronectin receptor, beta polypeptide, antigen CD29 includes MDF2, MSK12) |
|  | P38646 | heat shock 70kDa protein 9 (mortalin) |
|  | P06748 | nucleophosmin 1 (nucleolar phosphoprotein B23, numatrin) pseudogene 21 |
|  | P24347 | matrix metallopeptidase 11 (stromelysin 3) |
|  | Q07954 | low density lipoprotein-related protein 1 (alpha-2-macroglobulin receptor) |
|  | P24821 | tenascin C |
|  | P26599 | polypyrimidine tract binding protein 1 |
|  | P36871 | phosphoglucomutase 1 |
|  | P55209 | nucleosome assembly protein 1-like 1 |
|  | Q14126 | desmoglein 2 |
|  | P11413 | glucose-6-phosphate dehydrogenase |
|  | Q9NS15 | latent transforming growth factor beta binding protein 3 |
|  | P52823 | stanniocalcin 1 |
|  | P19338 | nucleolin |
|  | O43570 | carbonic anhydrase XII |
|  | P33908 | mannosidase, alpha, class 1A, member 1 |
|  | Q96TA1 | family with sequence similarity 129, member B |
|  | P08581 | met proto-oncogene (hepatocyte growth factor receptor) |
|  | P12955 | peptidase D |
|  | O75635 | serpin peptidase inhibitor, clade B (ovalbumin), member 7 |
|  | P17931 | lectin, galactoside-binding, soluble, 3 |
|  | P46926 | glucosamine-6-phosphate deaminase 1 |
|  | P20618 | proteasome (prosome, macropain) subunit, beta type, 1 |
|  | P40121 | capping protein (actin filament), gelsolin-like |
|  | P30043 | biliverdin reductase B (flavin reductase (NADPH)) |
|  | Q8NBJ7 | sulfatase modifying factor 2 |
|  | Q16706 | mannosidase, alpha, class 2A, member 1 |
|  | O95336 | 6-phosphogluconolactonase |
|  | P28300 | lysyl oxidase |
|  | P51858 | hepatoma-derived growth factor (high-mobility group protein 1-like) |
|  | P07942 | laminin, beta 1 |
|  | P09104 | enolase 2 (gamma, neuronal) |
|  | O15240 | VGF nerve growth factor inducible |
|  | Q92743 | HtrA serine peptidase 1 |
|  | Q8NBJ4 | golgi membrane protein 1 |
|  | P07225 | protein S (alpha) |
|  | P62805 | histone cluster 1, (H4l, H4k, H4h, H4j, H4i, H4d,H4c, H4f, H4e, H4b, H4a); cluster 2, (H4a, H4b); cluster 4, H4 |
|  | P05387 | ribosomal protein, large, P2 pseudogene 3; ribosomal protein, large, P2 |
|  | P22061 | protein-L-isoaspartate (D-aspartate) O-methyltransferase |
|  | P49327 | fatty acid synthase |
|  | P31939 | 5-aminoimidazole-4-carboxamide ribonucleotide formyltransferase/IMP cyclohydrolase |
|  | P30040 | endoplasmic reticulum protein 29 |
|  | O43505 | UDP-GlcNAc:betaGal beta-1,3-N-acetylglucosaminyltransferase 1; transferase 2 |
|  | P48637 | glutathione synthetase |
|  | P48307 | tissue factor pathway inhibitor 2 |
|  | O60462 | neuropilin 2 |
|  | P08254 | matrix metallopeptidase 3 (stromelysin 1, progelatinase) |
|  | Q9NRN5 | olfactomedin-like 3 |
|  | Q16719 | kynureninase (L-kynurenine hydrolase) |
|  | P52799 | ephrin-B2 |
|  | Q14766 | latent transforming growth factor beta binding protein 1 |
|  | Q14103 | heterogeneous nuclear ribonucleoprotein D (AU-rich element RNA binding protein 1, 37kDa) |
|  | Q9UKK9 | nudix (nucleoside diphosphate linked moiety X)-type motif 5 |
|  | P55083 | microfibrillar-associated protein 4 |
|  |  |  |
| **Exclusive proteins from MSC (n=14)** | **UniProt** | **Protein name** |
|  | Q99439 | calponin 2 |
|  | P08476 | inhibin, beta A |
|  | P37837 | transaldolase 1 |
|  | Q9UL46 | proteasome (prosome, macropain) activator subunit 2 (PA28 beta) |
|  | O15144 | actin related protein 2/3 complex, subunit 2, 34kDa |
|  | Q92626 | peroxidasin homolog (Drosophila) |
|  | Q96FW1 | OTU domain, ubiquitin aldehyde binding 1 |
|  | P35556 | fibrillin 2 |
|  | P09493 | tropomyosin 1 (alpha) |
|  | Q9Y696 | chloride intracellular channel 4 |
|  | Q9Y5Z4 | heme binding protein 2 |
|  | Q04917 | tyrosine 3-monooxygenase/tryptophan 5-monooxygenase activation protein, eta polypeptide |
|  | O95497 | vanin 1 |
|  | O95433 | AHA1, activator of heat shock 90kDa protein ATPase homolog 1 (yeast) |
|  |  |  |
| **Exclusive proteins from co-culture (n=10)** | **UniProt** | **Protein name** |
|  | Q12907 | lectin, mannose-binding 2 |
|  | O14773 | tripeptidyl peptidase I |
|  | Q9NY33 | dipeptidyl-peptidase 3 |
|  | Q9BTY2 | fucosidase, alpha-L- 2, plasma |
|  | Q99715 | collagen, type XII, alpha 1 |
|  | Q02952 | A kinase (PRKA) anchor protein 12 |
|  | P35080 | profilin 2 |
|  | P31150 | GDP dissociation inhibitor 1 |
|  | O60888 | cutA divalent cation tolerance homolog (E. coli) |
|  | Q14247 | Cortactin |
|  |  |  |
| **Exclusive proteins from U87MG (n=62)** | **UniProt** | **Protein name** |
|  | Q12905 | interleukin enhancer binding factor 2, 45kDa |
|  | P49746 | thrombospondin 3 |
|  | Q9BR76 | coronin, actin binding protein, 1B |
|  | P49321 | nuclear autoantigenic sperm protein (histone-binding) |
|  | P17900 | GM2 ganglioside activator |
|  | P54819 | adenylate kinase 2 |
|  | P38606 | ATPase, H+ transporting, lysosomal 70kDa, V1 subunit A |
|  | P05455 | Sjogren syndrome antigen B (autoantigen La) |
|  | P05452 | C-type lectin domain family 3, member B |
|  | P50502 | similar to heat shock 70kD protein binding protein; suppression of tumorigenicity 13 (colon carcinoma) |
|  | Q9UQ80 | proliferation-associated 2G4, 38kDa; proliferation-associated 2G4 pseudogene 4 |
|  | P10768 | esterase D/formylglutathione hydrolase |
|  | Q9NP97 | dynein, light chain, roadblock-type 1 |
|  | Q02388 | collagen, type VII, alpha 1 |
|  | P49588 | alanyl-tRNA synthetase |
|  | P17987 | hypothetical gene supported by BC000665; t-complex 1 |
|  | Q9Y6N7 | roundabout, axon guidance receptor, homolog 1 (Drosophila); similar to roundabout 1 isoform b |
|  | Q04446 | glucan (1,4-alpha-), branching enzyme 1 |
|  | Q9Y5F6 | protocadherin gamma subfamily C, 3; C, 5; C, 4; protocadherin gamma subfamily A, 12 |
|  | O00560 | syndecan binding protein (syntenin) |
|  | Q6UVK1 | chondroitin sulfate proteoglycan 4 |
|  | Q99880 | histone cluster 1, H2bl |
|  | P11940 | poly(A) binding protein, cytoplasmic pseudogene 5; poly(A) binding protein, cytoplasmic 1 |
|  | Q04837 | single-stranded DNA binding protein 1 |
|  | O00764 | pyridoxal (pyridoxine, vitamin B6) kinase |
|  | O60687 | sushi-repeat-containing protein, X-linked 2 |
|  | P16949 | stathmin 1 |
|  | O95782 | adaptor-related protein complex 2, alpha 1 subunit |
|  | P00749 | plasminogen activator, urokinase |
|  | O76003 | glutaredoxin 3 |
|  | Q14914 | prostaglandin reductase 1 |
|  | Q14956 | glycoprotein (transmembrane) nmb |
|  | P01137 | transforming growth factor, beta 1 |
|  | P56537 | eukaryotic translation initiation factor 6 |
|  | P10646 | tissue factor pathway inhibitor (lipoprotein-associated coagulation inhibitor) |
|  | P40227 | chaperonin containing TCP1, subunit 6A (zeta 1) |
|  | Q9Y2B0 | canopy 2 homolog (zebrafish) |
|  | P15586 | glucosamine (N-acetyl)-6-sulfatase |
|  | P41222 | prostaglandin D2 synthase, hematopoietic; prostaglandin D2 synthase 21kDa (brain) |
|  | O95084 | protease, serine, 23 |
|  | Q13162 | peroxiredoxin 4 |
|  | Q86VP6 | cullin-associated and neddylation-dissociated 1 |
|  | P43490 | nicotinamide phosphoribosyltransferase |
|  | P00751 | complement factor B |
|  | P00492 | hypoxanthine phosphoribosyltransferase 1 |
|  | P45974 | ubiquitin specific peptidase 5 (isopeptidase T) |
|  | P08833 | insulin-like growth factor binding protein 1 |
|  | O75882 | attractin |
|  | P49773 | histidine triad nucleotide binding protein 1 |
|  | Q13509 | tubulin, beta 3; melanocortin 1 receptor (alpha melanocyte stimulating hormone receptor) |
|  | P78559 | microtubule-associated protein 1A |
|  | P04062 | glucosidase, beta; acid (includes glucosylceramidase) |
|  | Q16363 | laminin, alpha 4 |
|  | P19367 | hexokinase 1 |
|  | P49368 | chaperonin containing TCP1, subunit 3 (gamma) |
|  | Q15435 | protein phosphatase 1, regulatory (inhibitor) subunit 7 |
|  | Q8IVF2 | AHNAK nucleoprotein 2 |
|  | Q96PD2 | discoidin, CUB and LCCL domain containing 2 |
|  | P07954 | fumarate hydratase |
|  | Q9NQC3 | reticulon 4 |
|  | Q9Y4L1 | hypoxia up-regulated 1 |
|  | Q6UXH9 | peptidase domain containing associated with muscle regeneration 1 |
