# Supplementary material for: Mesenchymal stem cells enhance tumorigenic properties of human glioblastoma through independent cell-cell communication mechanisms
Source: Oncotarget. 2018 May 15;9(37):24766–77. doi: 10.18632/oncotarget.25346 (PMC5973871; doi:10.18632/oncotarget.25346)
Supplement: Supplementary file 3 [file oncotarget-09-24766-s003.docx]

Supplementary Table 3: Exclusive and differentially expressed (over or hipoexpressed) proteins identified by *LC-MS/MS* in conditioned medium from U87MG + MSC co-culture, as compared with controls (MSC and U87MG single cultures).

| **UniProt** | **Exclusive proteins from cell co-culture** |
| --- | --- |
| Q12907 | lectin, mannose-binding 2 |
| O14773 | tripeptidyl peptidase I |
| Q9NY33 | dipeptidyl-peptidase 3 |
| Q9BTY2 | fucosidase, alpha-L- 2, plasma |
| Q99715 | collagen, type XII, alpha 1 |
| Q02952 | A kinase (PRKA) anchor protein 12 |
| P35080 | profilin 2 |
| P31150 | GDP dissociation inhibitor 1 |
| O60888 | cutA divalent cation tolerance homolog (E. coli) |
| Q14247 | cortactin |
| **UniProt** | **Overexpressed proteins from cell co-culture** |
| Q9ULV4 | coronin, actin binding protein, 1C |
| P07737 | profilin 1 |
| P15311 | hypothetical protein LOC100129652; ezrin |
| P05121 | serpin peptidase inhibitor, clade E/ nexin, plasminogen activator inhibitor type 1 |
| Q9C0K3 | actin-related Arp11 |
| O43707 | actinin, alpha 4 |
| P16930 | fumarylacetoacetate hydrolase (fumarylacetoacetase) |
| P28066 | proteasome (prosome, macropain) subunit, alpha type, 5 |
| P26038 | moesin |
| O75368 | SH3 domain binding glutamic acid-rich protein like |
| Q92520 | family with sequence similarity 3, member C |
| O75369 | filamin B, beta (actin binding protein 278) |
| P35579 | myosin, heavy chain 9, non-muscle |
| O60888 | cutA divalent cation tolerance homolog (E. coli) |
| P35580 | myosin, heavy chain 10, non-muscle |
| P13693 | similar to tumor protein, translationally-controlled 1 |
| Q15149 | similar to Plectin 1 (PLTN) (PCN) (Hemidesmosomal protein 1) (HD1) |
| P06753 | tropomyosin 3 |
| Q7Z406 | myosin, heavy chain 14 |
| P60900 | proteasome (prosome, macropain) subunit, alpha type, 6 |
| P61158 | ARP3 actin-related protein 3 homolog (yeast) |
| P13611 | versican |
| Q9BQ16 | sparc/osteonectin, cwcv and kazal-like domains proteoglycan (testican) 3 |
| P25786 | proteasome (prosome, macropain) subunit, alpha type, 1 |
| P25787 | proteasome (prosome, macropain) subunit, alpha type, 2 |
| O75635 | serpin peptidase inhibitor, clade B (ovalbumin), member 7 |
| P27797 | calreticulin |
| P35609 | actinin, alpha 2 |
| P62158 | calmodulin 3 (phosphorylase kinase, delta); calmodulin 2; calmodulin 1 |
| **UniProt** | **Overexpressed proteins from cell co-culture** |
| P12814 | actinin, alpha 1 |
| P00491 | nucleoside phosphorylase |
| P61160 | ARP2 actin-related protein 2 homolog (yeast) |
| P58107 | epiplakin 1 |
| P68032 | actin, alpha, cardiac muscle 1 |
| P55072 | valosin-containing protein |
| P35749 | myosin, heavy chain 11, smooth muscle |
| Q13219 | PAPPA antisense RNA; pregnancy-associated plasma protein A, pappalysin 1 |
| Q08043 | actinin, alpha 3 |
| Q01518 | CAP, adenylate cyclase-associated protein 1 (yeast) |
| Q96D15 | reticulocalbin 3, EF-hand calcium binding domain |
| P35237 | serpin peptidase inhibitor, clade B (ovalbumin), member 6 |
| P09238 | matrix metallopeptidase 10 (stromelysin 2) |
| Q9P1U1 | ARP3 actin-related protein 3 homolog B (yeast) |
| P50395 | GDP dissociation inhibitor 2 |
| P23284 | peptidylprolyl isomerase B (cyclophilin B) |
| Q16658 | fascin homolog 1, actin-bundling protein (Strongylocentrotus purpuratus) |
| Q9UNN8 | protein C receptor, endothelial (EPCR) |
| P08254 | matrix metallopeptidase 3 (stromelysin 1, progelatinase) |
| Q08629 | sparc/osteonectin, cwcv and kazal-like domains proteoglycan (testican) 1 |
| P68133 | actin, alpha 1, skeletal muscle |
| P21810 | biglycan |
| Q13740 | hypothetical protein activated leukocyte cell adhesion molecule |
| **UniProt** | **Hipoexpressed proteins from cell co-culture** |
| P07355 | annexin A2 pseudogene 3; annexin A2; annexin A2 pseudogene 1 |
| P17655 | calpain 2, (m/II) large subunit |
| P78417 | glutathione S-transferase omega 1 |
| P19021 | peptidylglycine alpha-amidating monooxygenase |
| P27816 | microtubule-associated protein 4 |
| P60709 | actin, beta |
| P08865 | ribosomal protein SA pseudogene 9; 8; 58; 19; 18; 15; 61; 29; 12 |
| P68371 | tubulin, beta 2C |
| P61604 | heat shock 10kDa protein 1 (chaperonin 10) |
| P16035 | TIMP metallopeptidase inhibitor 2 |
| P55786 | hypothetical protein FLJ11822; aminopeptidase puromycin sensitive |
| Q9BYX7 | POTE ankyrin domain family, member K |
| Q14194 | collapsin response mediator protein 1 |
| Q99497 | Parkinson disease (autosomal recessive, early onset) 7 |
| P14649 | myosin, light chain 6B, alkali, smooth muscle and non-muscle |
| Q06830 | peroxiredoxin 1 |
| A5A3E0 | POTE ankyrin domain family, member F |
| P68104 | eukaryotic translation elongation factor 1 alpha-like 7;3; 1; alpha 1 |
| Q05639 | eukaryotic translation elongation factor 1 alpha 2 |
| P04083 | annexin A1 |
| P18669 | phosphoglycerate mutase 1 (brain) |
| **UniProt** | **Hipoexpressed proteins from cell co-culture** |
| P22314 | ubiquitin-like modifier activating enzyme 1 |
| P60903 | S100 calcium binding protein A10 |
| P68363 | hypothetical gene supported by AF081484; NM_006082; tubulin, alpha 1b |
| P80303 | nucleobindin 2 |
| P41250 | glycyl-tRNA synthetase |
| P41219 | peripherin |
| Q58FF3 | heat shock protein 90kDa beta (Grp94), member 2 (pseudogene) |
| P09972 | aldolase C, fructose-bisphosphate |
| P55290 | cadherin 13, H-cadherin (heart) |
| P14550 | aldo-keto reductase family 1, member A1 (aldehyde reductase) |
| P15259 | phosphoglycerate mutase 2 (muscle) |
| P24534 | eukaryotic translation elongation factor 1 beta 2 and factor 1 beta 2-like |
| P46821 | microtubule-associated protein 1B |
| P35442 | thrombospondin 2 |
| P02751 | fibronectin 1 |
| Q8N0Y7 | phosphoglycerate mutase family member 4 |
| Q99536 | vesicle amine transport protein 1 homolog (T. californica) |
| P80723 | brain abundant, membrane attached signal protein 1 |
| O00469 | procollagen-lysine, 2-oxoglutarate 5-dioxygenase 2 |
| P04075 | aldolase A, fructose-bisphosphate |
| Q15121 | phosphoprotein enriched in astrocytes 15 |
| P17936 | insulin-like growth factor binding protein 3 |
| P16152 | carbonyl reductase 1 |
| Q16352 | internexin neuronal intermediate filament protein, alpha |
| Q16555 | dihydropyrimidinase-like 2 |
| P13667 | protein disulfide isomerase family A, member 4 |
| Q16610 | extracellular matrix protein 1 |
| P30044 | peroxiredoxin 5 |
| P22392 | non-metastatic cells 1, protein (NM23A) |
| P14625 | heat shock protein 90kDa beta (Grp94), member 1 |
| P08670 | vimentin |
| Q09666 | AHNAK nucleoprotein |
| P09382 | lectin, galactoside-binding, soluble, 1 |
| P63104 | tyrosine 3-monooxygenase/tryptophan 5-monooxygenase activation protein |
| Q5VTE0 | eukaryotic translation elongation factor 1 alpha-like 7; 3; alpha 1 |
| P12110 | collagen, type VI, alpha 2 |
| Q14112 | nidogen 2 (osteonidogen) |
| P07197 | neurofilament, medium polypeptide |
| P60660 | myosin, light chain 6, alkali, smooth muscle and non-muscle |
| P07196 | neurofilament, light polypeptide |
| P35555 | fibrillin 1 |
| Q15582 | transforming growth factor, beta-induced, 68kDa |
| Q9UI42 | carboxypeptidase A4 |
| Q9H853 | tubulin, alpha 4b (pseudogene) |
| P26641 | eukaryotic translation elongation factor 1 gamma |
| **UniProt** | **Hipoexpressed proteins from cell co-culture** |
| O43854 | EGF-like repeats and discoidin I-like domains 3 |
| P35754 | glutaredoxin (thioltransferase) |
| O75874 | isocitrate dehydrogenase 1 (NADP+), soluble |
| P26022 | pentraxin-related gene, rapidly induced by IL-1 beta |
| O60664 | mannose-6-phosphate receptor binding protein 1 |
| P63261 | actin, gamma 1 |
| Q6S8J3 | POTE ankyrin domain family, member E |
| P23381 | tryptophanyl-tRNA synthetase |
